# Sense of taste in patients after cochlear implantation-preliminary study

**DOI:** 10.4314/ahs.v21i4.37

**Published:** 2021-12

**Authors:** Piotr H Skarzynski, Marcin Wojciechowski, Magdalena B Skarzynska, Piotr Fronczak

**Affiliations:** 1 Institute of Sensory Organs, Kajetany, Poland; 2 Teleaudiology and Screening Department, World Hearing Center, Institute of Physiology and Pathology of Hearing, Warsaw/Kajetany, Poland; 3 Heart Failure and Cardiac Rehabilitation Department, Faculty of Medicine, Medical University of Warsaw, Warsaw, Poland; 4 Oto-Rhino-Laryngology Surgery Clinic, World Hearing Center, Institute of Physiology and Pathology of Hearing, Warsaw/Kajetany, Poland; 5 Center of Hearing and Speech Medincus, Kajetany, Poland

**Keywords:** Sense of taste, taste disorders, cochlear implant surgery, quality of life, partial deafness treatment

## Abstract

**Background:**

Taste is the leading sense in how we determine the quality of consumed food. Proper gustatory sensation largely determines the well-being and health of an organism, and this affects their quality of life.

**Objectives:**

The aim of the present study was to estimate the risk of early taste disorders following implantation surgery.

**Methods:**

Twenty patients underwent a taste test before, 1 day after, and 1 month after cochlear implantation. The taste sensations of sweet, sour, salty, and bitter were determined.

**Results:**

Statistical analysis showed no significant differences (p > 0.05) between individual tests among the entire study group. After dividing the respondents into smoking (n=6) and non-smoking (n=14) groups, only a weak correlation (p = 0.043) was found between the results of the first and second examination in the smoker group. However, a statistically significant decrease in the number of saline-sensitive (p<0.001) and acid-sensitive (p = 0.042) subjects was observed.

**Conclusion:**

These findings suggest that people after a cochlear implant may have transient taste disorders. Taste disorder called dysgeusia may be an early complication after the implantation procedure contributing to deterioration of patients quality of life.

## Introduction

Taste is the leading sense in how we determine the quality of consumed food. It enables us to differentiate food, and so allows us to avoid consuming rotten products or toxic substances. Proper gustatory sensation largely determines the well-being and health of an organism, and this affects their quality of life. Abnormal gustatory sensation may result in reduced appetite, weight loss, and malnutrition^1^–^3^.

Many prescribed medications such as over-the-counter (OTC) or herbal drugs, vitamins, and minerals may cause taste malfunction or taste disturbance, although the mechanism is unclear. On the one hand, there is a lack of reliable clinical trials and research, and on the other hand, patients sometimes do not link taste disturbances with the drugs they take^4^. Table 1 lists medications which may cause dysgeusia.

**Table 1:** Main groups of drugs (including examples) and chemical substances which may cause dysgeusia. Own work based on other studies^4^,^5^

**ACE INHIBITORS** (captopril)	**SARTANS** (losartan)
**DIURETICS** (acetazolamide)	**HISTAMINE ANTAGONISTS**
**CA-BLOCKERS** (amlodipine, diltiazem, nifedipine)	**CHEMOTHERAPEUTIC AGENTS**
**ANTIBIOTICS** (ampicillin, ethambutol, sulfamethoxazole, pentamidine, tetracycline)	**ANTIPSYCHOTICS** (risperidone, olanzapine, haloperidol)
**ANTIVIRAL DRUGS** (acyclovir)	**ANTIFUNGAL DRUGS** (terbinafine)
**ANTIDEPRESSANTS** (tricyclic antidepressants, serotonin reuptake inhibitors)	**LITHIUM**
**STATINS**	**ANXIOLYTIC AND HYPNOTIC DRUGS** (benzodiazepines)

Gustatory sensation may also be affected by chemoand radiotherapy, smoking, and the consumption of ethanol, as well as by various diseases^5^–^7^. The gustatory pathway passes near the ear, which predisposes patients with ENT problems to taste disorders.

Otorhinolaryngologic problems can arise from pathology of the oral mucosa, glossitis, nasal polyps, disorders of the salivary glands, and nerve damage during tonsillectomy^8^. A particular site of damage is the chorda tympani, which intersects the tympanic cavity and crosses between the auditory ossicles. Damage to it may occur in the course of disease of the middle ear^9^ or from otosurgical procedures^10^,^11^. Frequently, damage occurs during surgical excision of cholesteatomas^11^; damage may also occur during exploratory tympanotomy, when after a Rosen's incision the chorda tympani is incorrectly dissected. Temporary dysgeusia may also arise from surgery when irrigation is performed incorrectly or for too long; in such a situation, the chorda tympani may be excessively moisturised and become dysfunctional. Finally, damage can also occur during cochlear implant surgery.

The cochlear implant is an electronic hearing prosthesis which electrically stimulates the auditory nerve endings directly, replacing the damaged cochlear receptor organ by inducing functional potentials in the nerve. Cochlear implants are used to treat profound hearing loss and deafness, including partial deafness and unilateral deafness^12^–^14^. If performed early enough in hearing-impaired children, the procedure permits their normal development and proper functioning^15^.

According to data from the European Association of Cochlear Implant Users in 2011, the number of implantees in Western Europe is 200 per one million residents, while in Eastern Europe the figure is 75 per one million^16^. The objective of this study was to investigate the incidence of early dysgeusia in patients who had undergone cochlear implant surgery.

## Materials and methods

The preliminary research was conducted among 29 patients World Hearing Center of the Institute of Physiology and Pathology of Hearing, Warsaw. Twenty of the 29 enrolled patients completed the study. The study group was selected using a nonprobabilistic sampling technique. Eligibility criteria were being qualified for cochlear implant surgery and age between 18 and 75 including patients with residual hearing with partial deafness^17^–^19^. All patients gave written informed consent to participate in the study, which was conducted in accordance with the Declaration of Helsinki for medical research involving human subjects and was approved by the local ethics committee of the Institute of Physiology and Pathology of Hearing (approval number IFPS: KB/02/2016).

A set of “Taste Strips” (Burghart Messtechnik GmbH, Wedel, Germany) was used according to the user's manual in the current study, as well as a survey questionnaire consisting of 13 questions about dysgeusia incidence, smoking, last dental appointment, and any past otolaryngological procedures or neurological problems.

**Figure 1:**
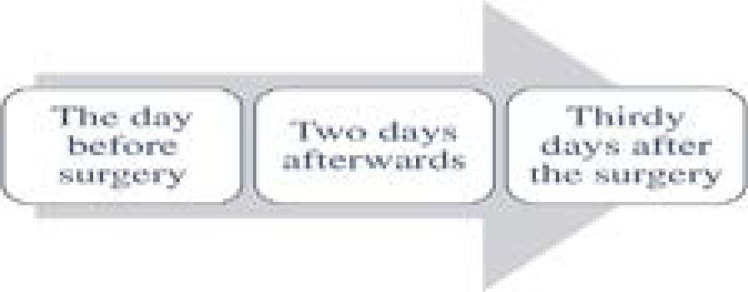
Chart of testing sessions.

There were 3 testing sessions, which were presented in the Figure 1. Testing with the Taste Strips was done according to the manufacturer's instructions included in the set. Patients were informed they could not eat, drink, or smoke for at least an hour before testing.

Testing started by presenting the patient with the taste of a paper strip not soaked in any test solution. Then, 4 strips were presented for each taste – sweet, salty, bitter, and sour – each of 4 different concentrations, resulting in a total of 16 strips in total. For each strip recognised correctly the patient scored 1 point. A negative test result (indicating no dysgeusia) required that the patient score at least 9 points overall, with at least 2 strips recognised for sweet, salty, and sour, plus one for bitter. Each participant used a bottle of low-mineral water to rinse theirtasting each strip. The order of presented strips was random.

Surgical procedure was performed according 6 step cochlear implantion technique dedicated for patients with residual hearing and partial deafness^20^. After conservative antromastoidotomy there was no exposure of chorda tympani when posterior tympanotomy was performed. During the surgery after drilling in all cases there was ringsing of tympanic cavity with dexamethasone solution (4mg/ml). It was applied after full perforamtion of posterior tympanotomy and taking out of bony overhang over round window. Rinsing solution was left behind for at least 5 minutes when bony bed on the cortical part for implant capsule was performed.

Statistical analysis was performed using Statistica 10 software by StatSoft. Results were evaluated using a Wilcoxon test for all subjects as well as for distinguishing smokers from non-smokers. In order to verify the relationships between the results, comparison of tests done at 2 days (test 2) and 30 days (test 3) was made. The significance level was p<0.05.

## Results

The study group comprised 20 subjects, including 8 women (40%) and 12 men (60%). The average age was 47.7 years. Almost half the patients (45%, n = 9) declared secondary education; 40%, higher education; and the others, vocational education.

Two subjects (10%) declared total pre-surgical hypogeusia, characterised by an altered gustatory sensation for food. The other respondents did not report any signs of dysgeusia, either recently or in the past.

### Testing with the “Taste Strips”

In the initial testing, nearly two-thirds of the patients (65%, n = 13) scored ≥ 9 points (mean score of 9.4), which, according to the manufacturer's instructions, indicates the absence of problems with sensing tastes. A slightly lower percentage of patients (60%, n = 12), recognised all tastes accurately.

The second testing showed that the number of the patients scoring ≥ 9 had increased to 75% (n = 15), and those who could accurately distinguish all tastes had increased to 65% (n = 13). We noticed improved scores in 2 from 6 patients who had been smokers but who had not smoked since the operation. After the surgery, one patient presented with a significant attenuation of all tastes, which persisted up to the third test (in sequence: 4 points, 4 points,9 points), and another who reported a constant sensation of salty taste in the mouth, which persisted up to the third test. The number of patients in the group with taste disorders at the second test was 5% (n = 1); the mean score was 10.

The third test showed that the number of the patients who scored ≥ 9 had decreased to 65% (n = 13), and the number who could accurately distinguish all tastes had decreased to 55% (n = 11). The results for the 2 smoking patients, who resumed smoking returned to the same level as before the operation (≤9). The mean score in the third test was 9.65. For further details, see table 2.

**Table 2:** Comparison of results of 3 testings with the “Taste Strips” set

	Test 1	Test 2	Test 3
Mean score	9.4	10	9.65
Patients scoring >=9 (%)	65	75	65
Patients identifying all tastes correctly (%)	60	65	55
Accurate interpretation of sweet taste (%)	90	95	95
Accurate interpretation of bitter taste (%)	85	85	85
Accurate interpretation of salty taste (%)	70	65	55
Accurate interpretation of sour taste (%)	90	80	65

Statistical analysis showed no significant differences (p >0.05) between the results of individual testings in the entire study group. The division into smokers (n=6) and non-smokers (n=14) revealed only a small correlation (p = 0.043) between the results of the first and second testing in the group of smokers. A statistically significant decrease in the number of patients accurately sensing salty (p <0.001) and sour (p = 0.04) was observed. For detailed information, see tables 3 and 4.

**Table 3:** Correlations between the results of individual testings in the entire study group, and non-smokers and smokers using a Wilcoxon test (*p*< 0.05)

	Entire group (*n* = 20)	Non-smokers (*n* = 14)	Smokers (*n* = 6)
Results: test 1 & test 2	0.1	0.59	0.043
Results: test 1 & test 3	0.43	0.42	0.89
Results: test 2 & test 3	0.42	0.40	0.079

**Table 4:** Correlations between tastes in Test 2 (*p*<0.05)

	χ^2^	df	*p*
Salty 3/Salty 2	10.48	1	0.001
Salty 3/Salty 1	13.16	1	0.0002
Sour 3/Sour 2	9.29	1	0.002
Sour 3/Sour 1	4.13	1	0.04

**Figure 2:**
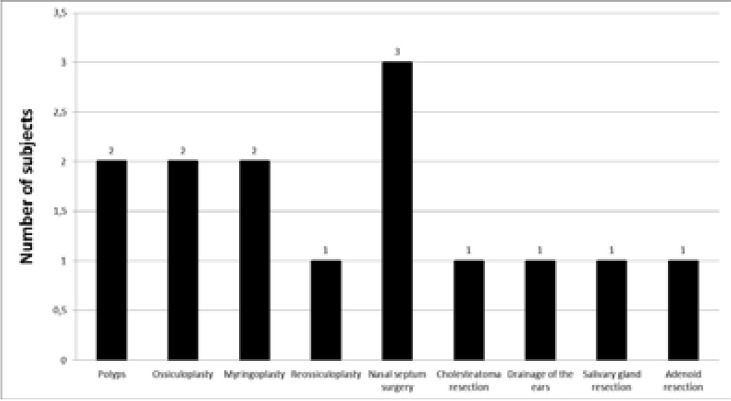
Types of otorhinolaryngological surgeries performed on the subjects before cochlear implant surgery.

Past head injuries were reported by 15% of the respondents (n = 3), and over a half of them (55%, n = 11) had undergone surgical or medical procedures. The number who had had otorhinolaryngological procedures ranged from 1 to 6. These were most often nasal septum surgeries. For details about the performed procedures, see Figure 2.

Only one patient had undergone a dental procedure a month before the surgery. The usage of prostheses or braces was reported by 25% of the patients (n = 5). Smoking was declared by 30% of the subjects (n = 6). The individual smoking timespan ranged from 2 to 40 years, with 10 to 60 cigarettes smoked per day. Some 35% of the subjects had been smokers 6 years or more ago.

**Figure 3:**
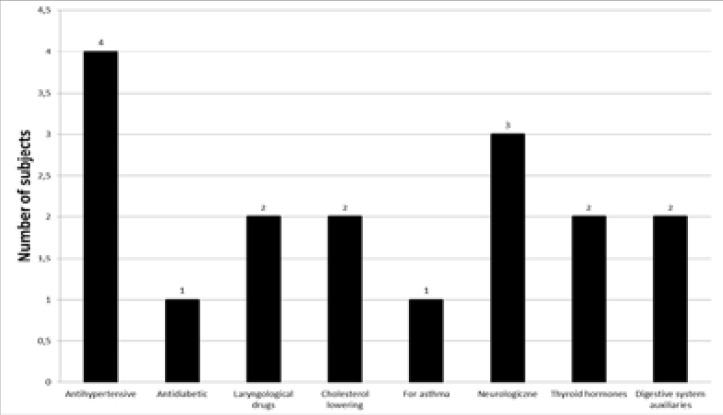
Groups of drugs taken by the subjects.

Drug use was indicated by almost half the patients (45%, n = 9). Most frequently these were antihypertensive drugs (20%, n = 4) and drugs for neurological diseases (15%, n = 3). The drugs used least often were those for asthma and diabetes. For details, see figure 3.

Exposure to chemicals at work was indicated by 15% of the subjects (n = 3). None of the patients had a history of any infection of the upper respiratory tract in the 1-month pre-surgical period.

The last two questions in the survey questionnaire considered the incidence of chronic diseases. Some 20% of the patients (n = 4) had undergone treatment for hypertension or neurological diseases.

**Figure 4:**
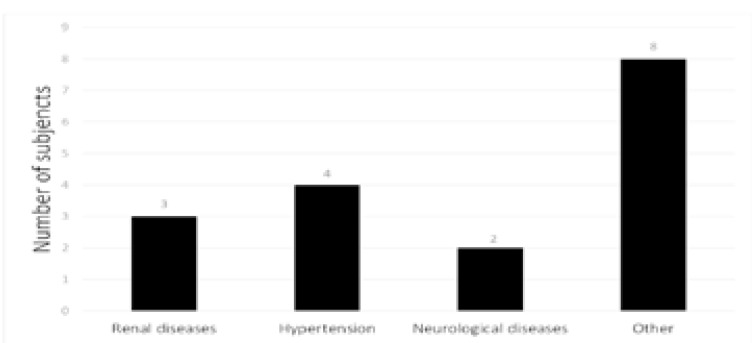
Types of chronic diseases reported by the subjects in the peri-surgical period.

A lesser number (15%, n = 3) had a history of renal diseases (Figure 4). Almost half the respondents (40%) indicated other ailments, the most frequent being gastric disease and thyroid conditions.

## Discussion

Abnormal gustatory sensations after cochlear implantation may be one of the post-surgical complications and may last even as long as a few months^21^. Published taste studies are rare, and mainly involve clinical trials in small groups. Most of the data is collected from observations and reports of patients at follow-up visits. As indicated in previous studies, taste disorders (dysgeusia) may manifest in different forms, not only in decreasing or intensifying taste sensitivity, but also, for example, in intolerance to the taste of a single product. In most cases, however, these abnormalities go away on their own.

The present study showed that the total taste score decreased significantly after surgery in 1 patient. These results are in line with those of Wagner et al. who carried out a study in 20 adults implanted bilaterally. The study used a questionnaire and testing was done with solutions of different flavour substances^22^. After the first surgery, one of the patients reported in the questionnaire that they suffered taste disorders, but these were not reflected in the results of testing. The number of subjects reporting gustatory sensation problems after the second surgery (implantation of the other ear) increased to 15%, but only in a single patient (5%) were these verified in tests with flavoured solutions. These results are in contrast to a studyy Mueller et al., where taste strip testing in 24 monaurally implanted patients revealed a statistically significant perceptual decrease in taste (via the tongue) on the side of the surgery (p = 0.004, a mean score of 10 vs. 8), with a statistically significant perceptual increase in taste (p = 0.037 and a mean score of 10 vs. 10.9) on the other side. Testing was done the day before and 4 days after surgery^23^. A follow-up testing was done 18 months after the surgery in 12 out of the 24 patients who had undergone the procedure. A further statistically significant perceptual attenuation of taste on the side of the surgery was observed (p = 0.015, mean score 7.7), as well as a statistically insignificant attenuation on the opposite side (p = 0.55, mean 9.2). Also in a study by Alzhrani et al. in a group of 26 implanted patients, post-surgical dysgeusia (3 days after surgery) was seen in a much higher percentage than in the current study, i.e. 19.2% of the subjects^24^. Follow-up testing 6 weeks after surgery did not show any gustatory sensation problems in any of the patients. It should be noted that the testing was performed at a centre where many cochlear implantations are routinely performed. A study by Jeppesen included 13 implanted patients^21^. Taste testing was done four times with the Taste Strips set, and subjective dysgeusia was measured with the use of a visual analog scale (VAS). Gustatory sensation attenuation was observed on the operated side (8.3 in the first study vs. 6.2 in a subsequent study) but it was not statistically significant. On the unoperated side, differences were also insignificant (7.2 in the 1st testing vs. 7.3 in the final testing). During the entire testing procedure, patients had significantly greater problems identifying salty and sour tastes, which is in line with the present study. In their subjective assessment, 23% of patients (3/13) reported post-surgical taste disorders.

There are also questionnaire studies in the literature about taste disorders after cochlear implantation^25^–^29^, but due to use of different methods than those used in the current study they are not discussed here.

The most important factor in loss of the taste is surgical approach. When there is situation that chorda tympani was exposed high speed drilling with diamond burr could influenced on taste. Even when chorda tympani was not exposed, heating from drilling during performation of posterior tympanotomy could affect taste. Another factor is to be as less invasive as possible. For example, in suprameatal approach there is higher probability for chorda tympani injury^25^,^30^. Long time of surgery and not enough proper irrigation can cause drying of chorda tympani what also could influence for taste disturbance.

## Conclusion

Dysgeusia is a possible early complication of cochlear implant surgery, occurring directly after the procedure and sometimes after a delay. It contributes to impairment of the quality of life of patients. These results suggest that people with a cochlear implant may have temporary taste disorders. Even though dysgeusia occurred at a moderate rate of incidence, the rate cannot be extended to a larger group of patients, and so further studies are necessary.
